# Cellular localization of the hybrid pyruvate/2-oxoglutarate dehydrogenase complex in the actinobacterium *Corynebacterium glutamicum*

**DOI:** 10.1128/spectrum.02668-23

**Published:** 2023-09-27

**Authors:** Lea Sundermeyer, Jan-Gerrit Folkerts, Benita Lückel, Christina Mack, Meike Baumgart, Michael Bott

**Affiliations:** 1 IBG-1: Biotechnology, Institute of Bio- and Geosciences, Forschungszentrum Jülich, Jülich, Germany; 2 Bioeconomy Science Center (BioSC), Forschungszentrum Jülich, Jülich, Germany; CNRS - University of Toulouse, Toulouse, France

**Keywords:** *Corynebacterium glutamicum*, 2-oxoglutarate dehydrogenase complex, pyruvate dehydrogenase complex, ODH inhibitory protein OdhI, cellular localization, fluorescence microscopy

## Abstract

For many bacterial proteins, specific localizations within the cell have been demonstrated, but enzymes involved in central metabolism are usually considered to be homogenously distributed within the cytoplasm. Here, we provide an example for a spatially defined localization of a unique enzyme complex found in actinobacteria, the hybrid pyruvate/2-oxoglutarate dehydrogenase complex (PDH-ODH). In non-actinobacterial cells, PDH and ODH form separate multienzyme complexes of megadalton size composed of three different subunits, E1, E2, and E3. The actinobacterial PDH-ODH complex is composed of four subunits, AceE (E1p), AceF (E2p), Lpd (E3), and OdhA (E1oE2o). Using fluorescence microscopy, we observed that in *Corynebacterium glutamicum*, all four subunits are co-localized in distinct spots at the cell poles, and in larger cells, additional spots are present at mid-cell. These results further confirm the existence of the hybrid complex. The unphosporylated OdhI protein, which binds to OdhA and inhibits ODH activity, was co-localized with OdhA at the poles, whereas phosphorylated OdhI, which does not bind OdhA, was distributed in the entire cytoplasm. Isocitrate dehydrogenase and glutamate dehydrogenase, both metabolically linked to ODH, were evenly distributed in the cytoplasm. Based on the available structural data for individual PDH-ODH subunits, a novel supramolecular architecture of the hybrid complex differing from classical PDH and ODH complexes has to be postulated. Our results suggest that localization at the poles or at mid-cell is most likely caused by nucleoid exclusion and results in a spatially organized metabolism in actinobacteria, with consequences yet to be studied.

**IMPORTANCE:**

Enzymes involved in the central metabolism of bacteria are usually considered to be distributed within the entire cytoplasm. Here, we provide an example for a spatially defined localization of a unique enzyme complex of actinobacteria, the hybrid pyruvate dehydrogenase/2-oxoglutarate dehydrogenase (PDH-ODH) complex composed of four different subunits. Using fusions with mVenus or mCherry and fluorescence microscopy, we show that all four subunits are co-localized in distinct spots at the cell poles, and in larger cells, additional spots were observed at mid-cell. These results clearly support the presence of the hybrid PDH-ODH complex and suggest a similar localization in other actinobacteria. The observation of a defined spatial localization of an enzyme complex catalyzing two key reactions of central metabolism poses questions regarding possible consequences for the availability of substrates and products within the cell and other bacterial enzyme complexes showing similar behavior.

## INTRODUCTION

Actinomycetes form a large and diverse bacterial class of Gram-positive bacteria (1) including human pathogens such as *Mycobacterium tuberculosis* and *Corynebacterium diphtheriae* as well as biotechnologically important members such as antibiotics-producing *Streptomcyces* species (2) or the amino acid producer *Corynebacterium glutamicum* (3 – 6). Therefore, actinobacterial metabolism and its regulation are subjects of ongoing research to identify novel targets for the treatment of the pathogens and the development of biotechnological producer strains. Previous studies have uncovered some unique features in the regulation and structural organization of the 2-oxoglutarate dehydrogenase complex (ODH) in actinobacteria.

ODH catalyzes the oxidative decarboxylation of 2-oxoglutarate to succinyl-coenzyme A (CoA) with concomitant reduction of NAD^+^ to NADH. It usually consists of three subunits, 2-oxoglutarate decarboxylase (E1o), dihydrolipoyl transsuccinylase (E2o), and dihydrolipoyl dehydrogenase (E3). E3 is shared with the pyruvate dehydrogenase complex (PDH), composed in addition of E1p (pyruvate decarboxylase) and E2p (dihydrolipoyl transacetylase) (7). In the native state, PDH and ODH form large complexes of several megadalton with a core made of the E2 component (8 – 11). *C. glutamicum* and many other actinobacteria do not possess a separate E2o subunit. Rather, the succinyl transferase domain of E2o is fused to the E1o subunit (12, 13). Since this unusual E1oE2o component only possesses the succinyl transferase domain but lacks the lipoyl-binding domains of regular E2o subunits, an interaction of corynebacterial E1oE2o with E2p is necessary, since the transfer of the succinyl group to CoA requires the lipoic acid residues.

In fact, purification of a Strep-tagged variant of E1oE2o (OdhA) from *C. glutamicum* revealed co-purification with E2p (AceF), E1p (AceE), and E3 (Lpd), and *vice versa*, purification of a Strep-tagged variant of E1p led to co-purification with E1oE2o, E2p, and E3 (14). Subsequent studies confirmed that the succinyl transferase domain of E1oE2o uses the lipoyl groups of E2p to transfer the succinyl group to CoA (15). Evidence for a hybrid PDH-ODH complex in *C. glutamicum* was also obtained in a recent biochemical study (16). Another unusual structural feature was unraveled for the E2p subunit AceF of *C. glutamicum* (17). The E2 subunits of most species form homo-trimers that further oligomerize by intermolecular trimer-trimer interactions mediated by a conserved C-terminal 3_10_ hydrophobic helix, leading to the assembly of 8 or even 20 trimers, depending on the species (18). The E2p protein of *C. glutamicum* also forms trimers, but a unique C-terminal helix bearing an actinobacteria-specific insertion precludes trimer oligomerization (17).

ODH activity is allosterically activated by acetyl-CoA (19, 20) and inhibited via protein-protein interaction with the small forkhead-associated domain-containing protein OdhI (14). OdhI binds with nanomolar affinity to the C-terminal 2-oxoglutarate decarboxylase domain of the E1oE2o subunit and inhibits ODH activity with an apparent *K*_i_ of 2.4 nM (14, 21, 22). The importance of OdhI for shifting carbon flux at the 2-oxoglutarate node from the tricarboxylic acid (TCA) cycle toward l-glutamate synthesis and thus nitrogen assimilation by the NADPH-dependent glutamate dehydrogenase was demonstrated by the fact that an *odhI* deletion mutant of *C. glutamicum* almost completely lost its ability for L-glutamate overproduction (23). Inhibition of ODH activity by OdhI is reversible and controlled by covalent modification of OdhI (14, 21, 22). Phosphorylation of the N-terminal Thr-14 residue of OdhI by the serine/threonine protein kinase (STPK) PknG leads to a conformational change of OdhI and thereby prevents its binding to OdhA (24). Dephosphorylation by the phospho-serine/threonine protein phosphatase Ppp leads to a reactivation of OdhI (23, 25). OdhI can also be phosphorylated by the three other STPKs present in *C. glutamicum*, PknA, PknB, and PknL, at least *in vitro* (25). *In vivo*, besides unphosphorylated and monophosphorylated OdhI, diphosphorylated OdhI was also detected (14, 25), and PknB was shown to phosphorylate OdhI on Thr-15 (24). The mycobacterial OdhI homolog GarA has an inhibitory effect not only on ODH but also on the NADH-dependent glutamate dehydrogenase (GDH), while glutamate synthase is activated by GarA (26 – 28). These and further studies underline the importance of the regulation of carbon flux at the 2-oxoglutarate node by GarA in *Mycobacterium*.

As the function of OdhI and GarA is determined by their phosphorylation status, it is important to understand the signals controlling phosphorylation and dephosphorylation. Whereas the regulation of the phosphatase activity (Ppp in *Corynebacterium*, PstP in *Mycobacterium*) is unknown, a regulatory cascade controlling PknG activity has been elucidated. Initial experiments on a *pknG* deletion mutant of *M. tuberculosis* revealed the accumulation of glutamate and glutamine, leading to the proposal that PknG mediates the transfer of signals sensing nutritional stress and translates them into metabolic adaptation (29). In addition, a *pknG* deletion mutant of *C. glutamicum* revealed a twofold increased cellular L-glutamate level and a growth defect on agar plates containing L-glutamine as sole carbon and nitrogen source, which is caused by inhibition of ODH by unphosphorylated OdhI (14). The defect in glutamine utilization was also observed for mutants lacking *glnX* and *glnH*, which are located immediately upstream of *pknG* in a conserved operon and encode an integral membrane protein and a periplasmic binding protein. The glutamine phenotype suggested that GlnH and GlnX are part of a signal transduction cascade controlling PknG activity (14). GlnH of *M. tuberculosis* was shown to bind L-aspartate and L-glutamate with high affinity (K_D_ of 5 µM and 15 µM, respectively), whereas GlnH of *C. glutamicum* has an about 100-fold lower affinity for these amino acids (30, 31). The involvement of GlnH and GlnX in the control of OdhI phosphorylation was experimentally confirmed in *C. glutamicum* (31). GlnX contains four transmembrane helices and two large periplasmic domains, with N- and C-terminus facing the cytoplasm. A structural model suggested that both periplasmic domains form four-helix bundles and represent a new type of four-helix bundle proteins with a tandem arrangement. GlnX is supposed to activate PknG when GlnH is present in the ligand-bound state, thereby shifting the 2-oxoglutarate flux into the TCA cycle and reducing glutamate synthesis (31).

To gain further insights into the properties of the hybrid PDH-ODH complex and its regulation by OdhI, we analyzed the intracellular localization and surprisingly discovered that unphosphorylated OdhI, its interaction partner OdhA, and the three other subunits of the hybrid PDH-ODH complex, AceE, AceF, and Lpd, are localized at the poles and in larger cells also at mid-cell, whereas phosphorylated OdhI, isocitrate dehydrogenase, and glutamate dehydrogenase were distributed in the entire cytoplasm. Further experiments led us to propose that this unexpected localization of the PDH-ODH complex is caused by nucleoid exclusion.

## RESULTS

### Localization of OdhI-mVenus and GarA-mVenus fusion proteins in *C. glutamicum*

**Fig 1:**
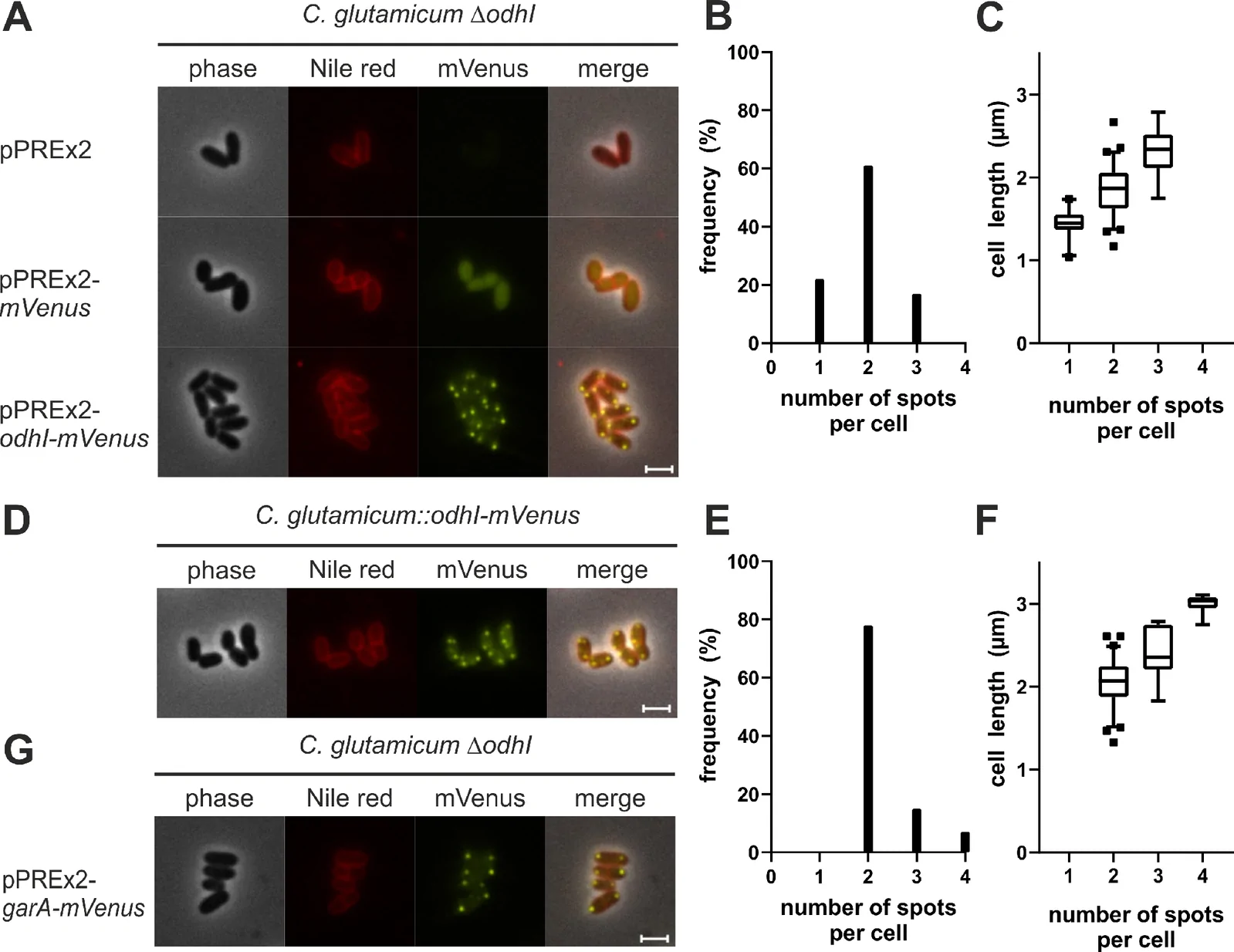
Intracellular localization of OdhI-mVenus in *C. glutamicum*. (**A**) Exemplary images of *C. glutamicum* ∆*odhI* cells carrying the plasmids pPREx2-*odhI-mVenus*, pPREx2-*mVenus*, or pPREx2. (**B**) Frequency of number of fluorescent spots per cell detected in *C. glutamicum* ∆*odhI* pPREx2-*odhI-mVenus*. (**C**) Correlation between the number of spots per cell from B and the length of the respective cell. (**D**) Exemplary images of OdhI-mVenus localization in a *C. glutamicum::odhI-mVenus* integration strain. (**E**) Frequency of number of fluorescent spots per cell detected in *C. glutamicum::odhI-mVenus*. (**F**) Correlation between the number of spots per cell from E and the length of the respective cell. (**G**) Exemplary images showing the localization of the OdhI homolog GarA in *C. glutamicum* ∆*odhI* pPREx2-*garA-mVenus*. The *C. glutamicum* strains were grown in CGXII medium with 2% (wt/vol) glucose. Cell membranes were stained using Nile red, and images were taken using an Axio imager M2 microscope. Scale bars in A, D, and G represent 2 µm. The line shown in C and F represents the median, and the box, the 25th to 75th percentile. The whiskers reach from 5th to 95th percentile. The results were obtained by analysis of 100 cells.

The cellular distribution of OdhI was initially analyzed with plasmid pPREx2-*odhI-mVenus* coding for a C-terminal fusion of OdhI with the yellow fluorescent protein mVenus under control of a P*_tac_* promoter. The plasmid was transferred into the *C. glutamicum* ∆*odhI* strain to prevent competition with native OdhI. Cells were cultivated in CGXII glucose minimal medium without induction and analyzed by fluorescence microscopy. Cell membranes were stained with Nile red to allow a better differentiation of individual cells. Contrary to our expectation to find OdhI-mVenus equally distributed throughout the cell, we surprisingly observed that most cells showed two bright fluorescent spots located at the cell poles. A weak fluorescence equally distributed within the entire cytoplasm was also visible (Fig. 1A). In the control strain transformed with pPREx2-*mVenus*, fluorescence was evenly distributed within the cytoplasm (Fig. 1A), whereas *C. glutamicum* ∆*odhI* carrying pPREx2 displayed no yellow fluorescence. These results suggested that the fluorescent polar spots are caused by a specific localization of OdhI. A statistical analysis of 100 cells revealed that the majority (61%) possessed two fluorescent spots, mostly located at the cell poles, while in 22% of the cells, only one spot was visible and 17% showed a third fluorescent spot, which was usually located at mid-cell (Fig. 1B). The cells showed the typical rod-shaped morphology of *C. glutamicum* with a mean length of 1.84 ± 0.38 µm. The number of OdhI-mVenus fluorescent spots positively correlated with the cell length (Fig. 1C).

To exclude that the polar localization of OdhI-mVenus is an artifact caused by unphysiologically high plasmid-based expression, we fused the genomically encoded *odhI* gene with *mVenus*, enabling native expression levels. Again, intense fluorescent spots were observed at the cell poles and at mid-cell of the integration strain *C. glutamicum::odhI-mVenus* (Fig. 1D). The strong fluorescence intensity of the spots suggested that the native *odhI* promoter is rather strong. The majority of cells contained two fluorescent spots (78%), and the residual cells possessed three or four spots. The number of spots per cell again positively correlated with the cell length (Fig. 1E and F). An increased mean cell length of the integration strain (2.17 ± 0.38 µm) might be caused by the absence of kanamycin and a plasmid.

Taken together, these results confirmed the formation of fluorescent OdhI-mVenus spots at the cell poles at physiological expression levels and showed that pPREx2-based *odhI-mVenus* expression is a suitable approach to study the localization of the fusion protein in different strain backgrounds. OdhI and its mycobacterial ortholog GarA share strong structural and functional similarities (13). Therefore, we tested if GarA behaves like OdhI with respect to its intracellular localization. In *C. glutamicum* ∆*odhI* transformed with pPREx2-*garA-mVenus*, the GarA-mVenus fusion protein also formed fluorescent spots at the cell poles (Fig. 1G). When expressed in *C. glutamicum* wild type (wt), fluorescent spots at the poles were almost absent, and GarA-mVenus fluorescence was evenly distributed in the entire cytoplasm (data not shown). This result can be explained by a significantly lower affinity of heterologous GarA to OdhA compared to the endogenous OdhI. In previous interaction studies by surface plasmon resonance experiments, a K_D_ value of 13.8 nM was determined for the binding of OdhI to OdhA (21), whereas for the binding of *Mycobacterium smegmatis* GarA to the OdhA-homolog KGD of *M. smegmatis* (58% sequence identity to OdhA), a K_D_ value of 1.92 µM was reported (32). Therefore, in *C. glutamicum* cells containing both OdhI and GarA-mVenus at comparable levels, OdhI would clearly outcompete GarA-mVenus for OdhA binding, thereby preventing the formation of fluorescent spots at the poles.

### Influence of serine/threonine protein kinases on OdhI-mVenus spot formation

**Fig 2:**
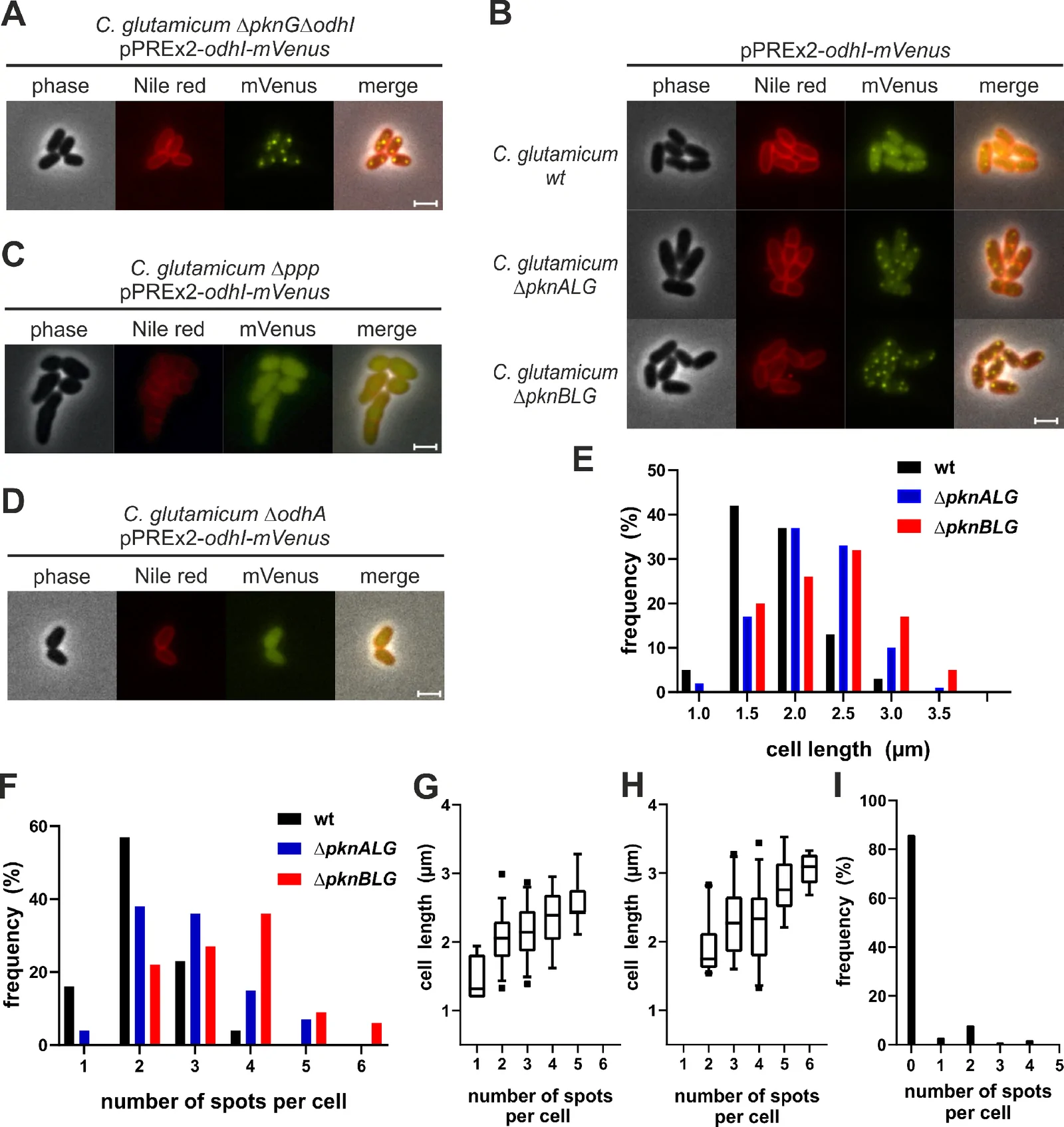
Influence of Ser/Thr protein kinases, the phosphatase Ppp, and the ODH subunit OdhA on the intracellular localization of OdhI-mVenus in *C. glutamicum*. (**A**) Exemplary images of *C. glutamicum* ∆*pknG*∆*odhI* pPREx2-*odhI-mVenus*. (**B**) Exemplary images of *C. glutamicum* wt, ∆*pknALG*, and ∆*pknBLG* carrying the expression plasmid pPREx2-*odhI-mVenus*. (**C**) Exemplary images of *C. glutamicum* ∆*ppp* pPREx2-*odhI-mVenus*. (**D**) Exemplary images of *C. glutamicum* ∆*odhA* pPREx2-*odhI-mVenus*. (**E**) Overview on the altered cell length of *C. glutamicum* ∆*pknALG* and ∆*pknBLG* compared to the wt. (**F**) Frequency of number of fluorescent spots per cell detected in *C. glutamicum* wt, ∆*pknALG*, and ∆*pknBLG*. (**G**) and (**H**) Correlation between the number of spots per cell and the length of the respective cell. Results of *C. glutamicum* ∆*pknALG* shown in G and results of *C. glutamicum* ∆*pknBLG* shown in H. (**I**) Frequency of number of fluorescent spots per cell in *C. glutamicum* ∆*ppp* pPREx2-*odhI-mVenus*. All strains were grown in CGXII medium with 2% (wt/vol) glucose. Cell membranes were stained using Nile red, and images were taken using an Axio imager M2 microscope. Scale bars in A, B, C, and D represent 2 µm. The line shown in G and H represents the median, and the box the 25th to 75th percentile. The whiskers reach from 5th to 95th percentile. The results were obtained by analysis of 100 cells per strain.

As the function of OdhI is dependent on its phosphorylation status, we analyzed the influence of the four STPKs PknA, PknB, PknG, and PknL on the localization of OdhI-mVenus using various deletion mutants transformed with pPREx2-*odhI-mVenus*. As shown in Fig. 2A, fluorescent spots were observed in *C. glutamicum* ∆*pknG*∆*odhI*. The spot numbers (24% of cells with one spot, 58% with two spots, 15% with three spots) were similar to the ones obtained for the ∆*odhI* strain, and a positive correlation with cell length (1.87 ± 0.41 µm on average) was observed. These results show that PknG is not required for the formation of the OdhI-mVenus spots. Besides PknG, the membrane-integral kinases PknA, PknB, and PknL, which are involved in the regulation of peptidoglycan synthesis (33 – 35), are also able to phosphorylate OdhI (24, 25). In *C. glutamicum*, peptidoglycan synthesis takes place at the cell poles and the septum (36), making the membrane-bound STPKs candidates for causing localization of OdhI at these sites. We investigated the strains *C. glutamicum* Δ*pknALG* and *C. glutamicum* Δ*pknBLG* lacking three of the four STPKs. A simultaneous deletion of *pknA* and *pknB* in *C. glutamicum* seems to be lethal (25). As the triple deletion strains still possess the genomic *odhI* gene, we used *C. glutamicum* wt transformed with pPREx2-*odhI-mVenus* as a control strain. Expression of *odhI-mVenus* led to the formation of fluorescent spots, both in the mutant strains and in the wt (Fig. 2B). While the wt possessed mainly two spots per cell (57%) and a mean cell length of 1.83 ± 0.40 µm, strain Δ*pknALG* showed an increased cell length (2.15 ± 0.44 µm) and an increased number of spots per cell (58% of cells containing three or more spots). These effects were even more prominent in the Δ*pknBLG* strain with a mean cell length of 2.30 ± 0.55 µm and 78% of cells possessing three or more spots (Fig. 2E and F). Again, a positive correlation between cell length and number of spots per cell was observed (Fig. 2G and H). Although at least one kinase is still present in the triple deletion mutants, the results clearly suggest that the STPKs are not involved in the spot formation of OdhI-mVenus. The higher number of spots per cell in the triple mutants correlates with their increased cell length but, in addition, might be due to a higher fraction of unphosphorylated OdhI (25). In this case, the results would suggest that PknB is more strongly involved in OdhI phosphorylation under the selected conditions than PknA, as much more cells had three OdhI spots in the absence of PknB than in the absence of PknA.

### Influence of the phospho-serine/threonine protein phosphatase Ppp on OdhI-mVenus spot formation

Next, we tested the influence of the absence of the membrane-bound phospho-serine/threonine protein phosphatase Ppp on the localization of OdhI-mVenus. Ppp was previously shown to dephosphorylate phosphorylated OdhI, and in a *C. glutamicum* Δ*ppp* strain, almost only phosphorylated OdhI is present (25). The deletion of *ppp* causes a strong growth defect and a strong alteration of the cell morphology, with rounded as well as prolonged cells (25). The absence of Ppp had a drastic effect on the localization of OdhI-mVenus. In the majority of Δ*ppp* cells, expression of *odhI-mVenus* did not lead to fluorescent spots, but rather to an equally distributed fluorescence signal throughout the cytoplasm. Fluorescent spots were only visible in ~14% of the cells, and in these cells, the number of spots varied between one and four and no specific localization was visible (Fig. 2C). This result strongly suggests that the fluorescent spots of OdhI-Venus are formed by unphosphorylated OdhI, but not by phosphorylated OdhI. This conclusion was further supported by the finding that plasmid-based synthesis of OdhI-mVenus variants, in which either one or both of the phosphorylated OdhI residues T14 and T15 were exchanged to alanine, also led to the formation of the fluorescent polar spots (Fig. S1). In addition, the higher number of OdhI spots in the triple deletion strains Δ*pknALG* and Δ*pknBLG* with increased levels of unphosphorylated OdhI fits with this conclusion.

### Influence of OdhA on OdhI-mVenus spot formation

As unphosphorylated OdhI was shown to be responsible for spot formation and OdhA is, at present, the only known interaction partner of OdhI in *C. glutamicum*, OdhA could cause the polar localization of OdhI-mVenus. We, therefore, constructed the deletion mutant *C. glutamicum* Δ*odhA* and transformed it with pPREx2-*odhI-mVenus*. No fluorescent spots at the poles or at mid-cell were visible anymore in this strain, and the cells showed an equally distributed fluorescence signal (Fig. 2D). This result confirmed that OdhA is required for the observed specific localization of OdhI.

### Localization of OdhA and co-localization with OdhI

**Fig 3:**
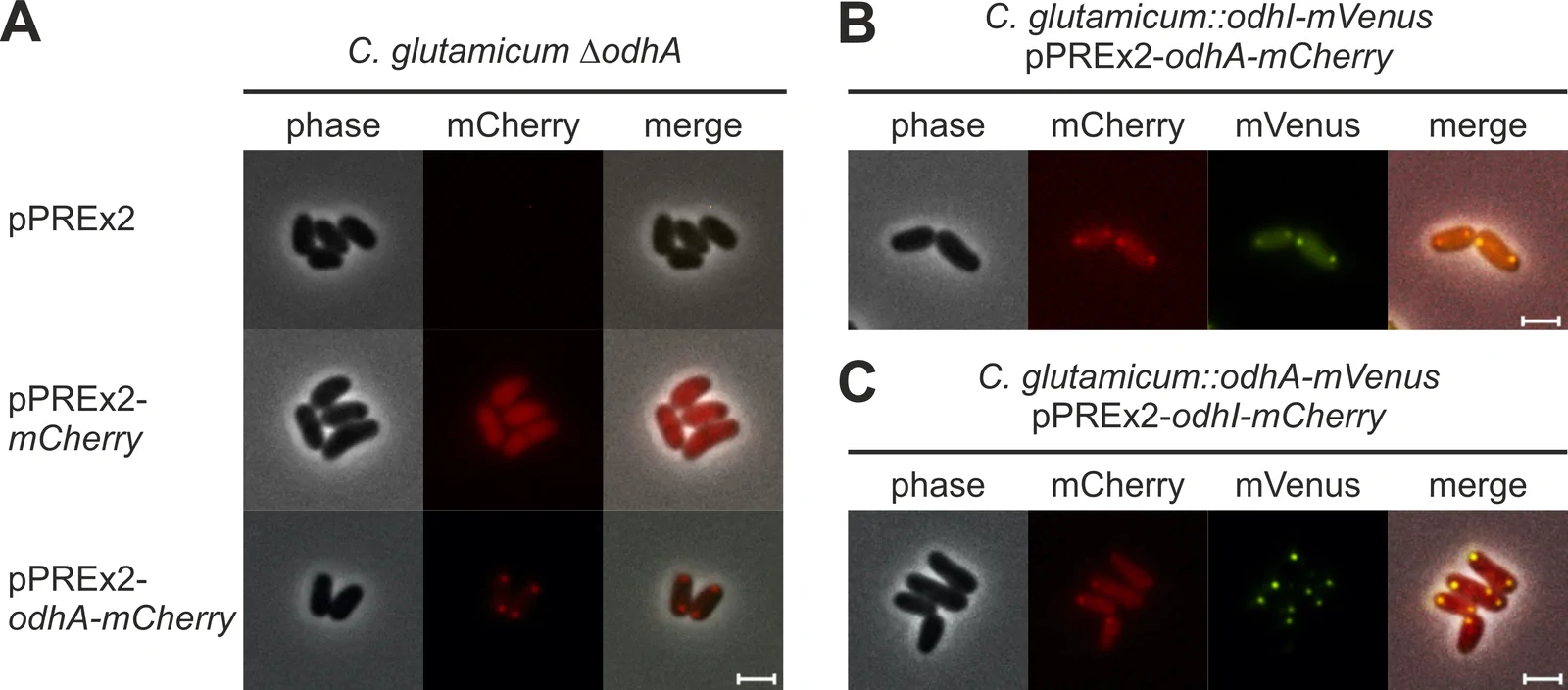
Localization of OdhA-mCherry and co-localization of OdhI and OdhA. (**A**) Exemplary images of *C. glutamicum* ∆*odhA* cells carrying the plasmids pPREx2-*odhA-mCherry*, pPREx2-*mCherry*, or pPREx2. (**B**) Exemplary images of *C. glutamicum::odhI-mVenus* pPREx2-*odhA-mCherry*. (**C**) Exemplary images of *C. glutamicum::odhA-mVenus* pPREx2-*odhI-mCherry*. The *C. glutamicum* strains were grown in CGXII medium with 2% (wt/vol) glucose, and images were taken using an Axio imager M2 microscope. Scale bars represent 2 µm.

To test if OdhA itself is also localized at the poles as suggested by the previous results, we constructed plasmid pPREx2-*odhA-mCherry* coding for OdhA with a C-terminal fusion to mCherry and analyzed the localization of the fusion protein in strain *C. glutamicum* ∆*odhA*. Due to the use of mCherry, membrane staining using Nile red was not possible in this experiment, but the cells could still be distinguished from each other. Similar to the results obtained for OdhI-mVenus, bright fluorescent spots located at the poles were observed in cells expressing *odhA-mCherry* (Fig. 3A). As controls, we also analyzed the fluorescence of *C. glutamicum* ∆*odhA* carrying pPREx2-*mCherry* to exclude artefacts caused by the fluorescent protein itself. As expected, expression of *mCherry* alone led to a strong, evenly distributed fluorescence in the entire cell (Fig. 3A). To test the co-localization of OdhI and OdhA, we used *C. glutamicum::odhI-mVenus* transformed with pPREx2-*odhA-mCherry*. Additionally, we constructed a *C. glutamicum::odhA-mVenus* integration strain and transformed it with pPREx2-*odhI-mCherry*. Both strains possessed mCherry and mVenus fluorescent spots at the poles and at mid-cell, which perfectly co-localized (Fig. 3B and C). This result further confirmed that unphosphorylated OdhI is located at the cell poles due to its binding to OdhA.

### Localization of other interaction partners of OdhA

**Fig 4:**
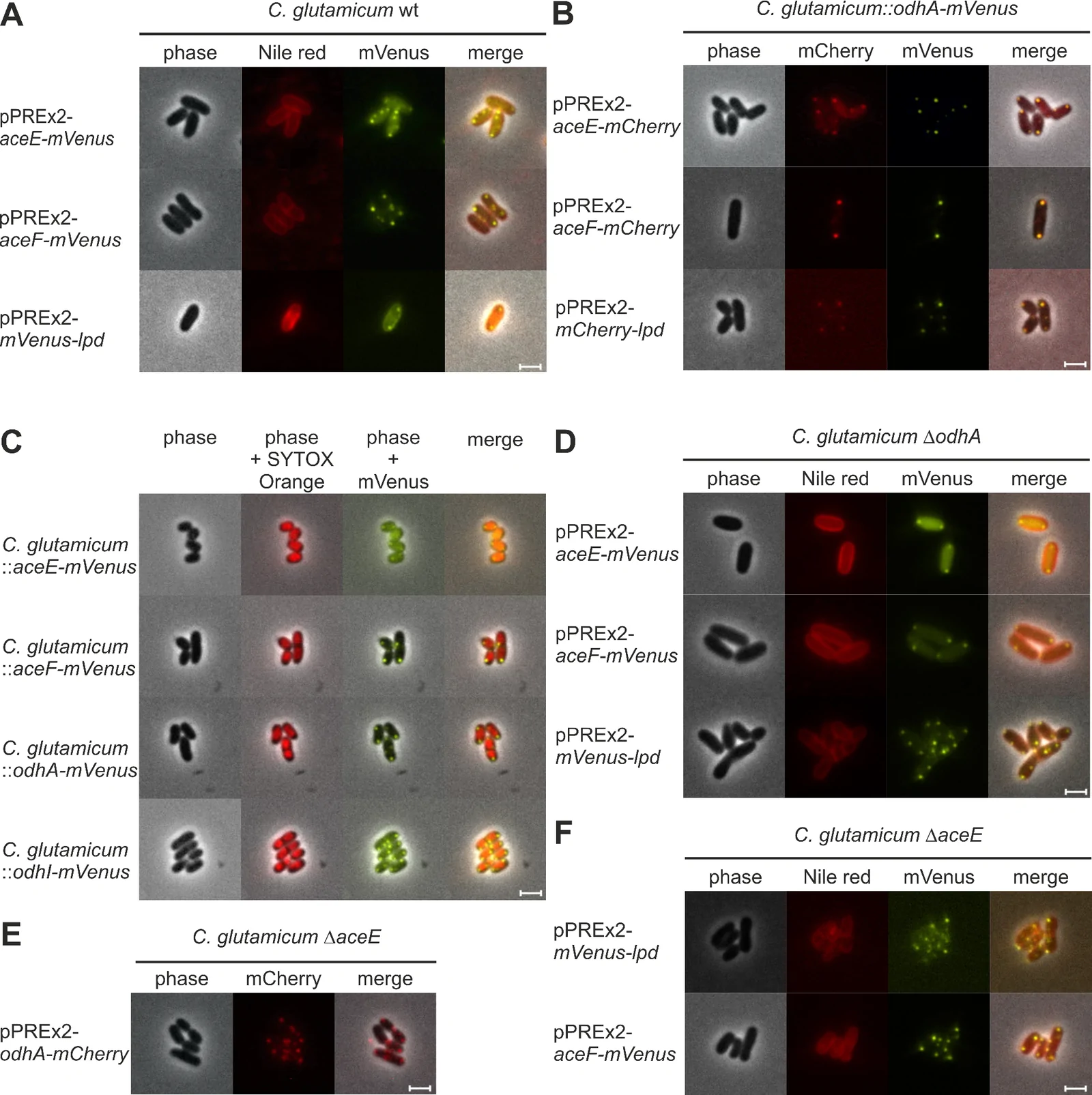
Localization of the PDH-ODH complex components in *C. glutamicum*. (**A**) Exemplary images of *C. glutamicum* wt cells carrying the expression plasmids pPREx2-*aceE-mVenus*, pPREx2-*aceF-mVenus*, or pPREx2*-mVenus-lpd*. (**B**) Exemplary images of *C. glutamicum::odhA-mVenus* pPREx2-*aceF-mCherry, C. glutamicum::odhA-mVenus* pPREx2-*aceE-mCherry,* and *C. glutamicum::odhA-mVenus* pPREx2*-mCherry-lpd*. (**C**) Exemplary images of *C. glutamicum::aceE-mVenus*, *C. glutamicum::aceF-mVenus*, *C. glutamicum::odhA-mVenus*, and *C. glutamicum::odhI-mVenus*. The DNA was stained using SYTOX Orange. (**D**) Exemplary images of *C. glutamicum* ∆*odhA* cells carrying the plasmids pPREx2-*aceF-mVenus*, pPREx2-*aceE-mVenus*, or pPREx2-*mVenus-lpd*. (**E**) Exemplary images of *C. glutamicum* ∆*aceE* pPREx2-*odhA-mCherry*. (**F**) Exemplary images of *C. glutamicum* ∆*aceE* cells carrying the plasmids pPREx2-*aceF-mVenus* and pPREx2*-mVenus-lpd*. All cells were grown in CGXII medium with 2% (wt/vol) glucose. In case of *C. glutamicum* ∆*aceE*, the medium was supplemented with 2 g L^−1^ acetate. If indicated, membranes were stained using Nile red, and images were taken using an Axio imager M2 microscope. Scale bars represent 2 µm.

As OdhA is part of the hybrid PDH-ODH complex, we next studied if the other three proteins of this complex also show a polar localization using plasmids coding for C-terminal fusions of AceE and AceF with mVenus and an N-terminal fusion of Lpd with mVenus. As shown in Fig. 4A, all three fusion proteins formed fluorescent spots in *C. glutamicum* wt, which mostly localized at the cell poles or at mid-cell. Notably, cells containing a C-terminal fusion of Lpd with mVenus still showed fluorescent spots, but also a strong evenly distributed fluorescence in the entire cytoplasm which was absent in the cells with the N-terminal fusion of Lpd with mVenus (Fig. S2). This result suggests that the C-terminal fusion might negatively affect the assembly of Lpd with the other subunits of the PDH-ODH complex. Furthermore, we analyzed the co-localization of the different PDH-ODH complex components using strain *C. glutamicum::odhA-mVenus* carrying either pPREx2-*aceE-mCherry*, pPREx2-*aceF-mCherry*, or pPREx2-*mCherry-lpd*. As shown in Fig. 4B, the OdhA-mVenus spots perfectly co-localized with the AceE-mCherry as well as with the AceF-mCherry and with the mCherry-Lpd spots. To further prove the localization of the entire PDH-ODH complex at the poles, we constructed the strains *C. glutamicum::aceE-mVenus* and *C. glutamicum::aceF-mVenus*. Also, these integration strains with native expression levels of *aceE* and *aceF* formed fluorescent spots at the poles, as observed for plasmid-based expression (Fig. 4C). DNA staining using SYTOX Orange showed that the fluorescent spots caused by the PDH-ODH complex components mainly localize in DNA free regions at the cell poles or at mid-cell (Fig. 4C).

### Relevance of OdhA and AceE for localization of the other subunits of the PDH-ODH complex

To test if the polar localization of the PDH-ODH complex is caused exclusively by only one of the four subunits, we planned to analyze mutant strains lacking *odhA*, *aceE*, *aceF*, or *lpd*. Whereas *∆odhA* and *∆aceE* strains were obtained, several attempts to construct ∆*aceF* and ∆*lpd* strains failed. The ∆*odhA* and ∆*aceE* strains showed severe growth defects as expected for cells lacking key enzymes of central metabolism. AceE-mVenus, AceF-Venus, and mVenus-Lpd still formed fluorescent spots at the poles in a Δ*odhA* deletion mutant (Fig. 4D), and OdhA-mCherry as well as AceF-mVenus and Lpd-mVenus formed polar spots in the Δ*aceE* deletion mutant (Fig. 4E and F). Therefore, neither OdhA nor AceE is required for polar localization of the other components of the complex.

### Localization of isocitrate dehydrogenase and glutamate dehydrogenase

**Fig 5:**
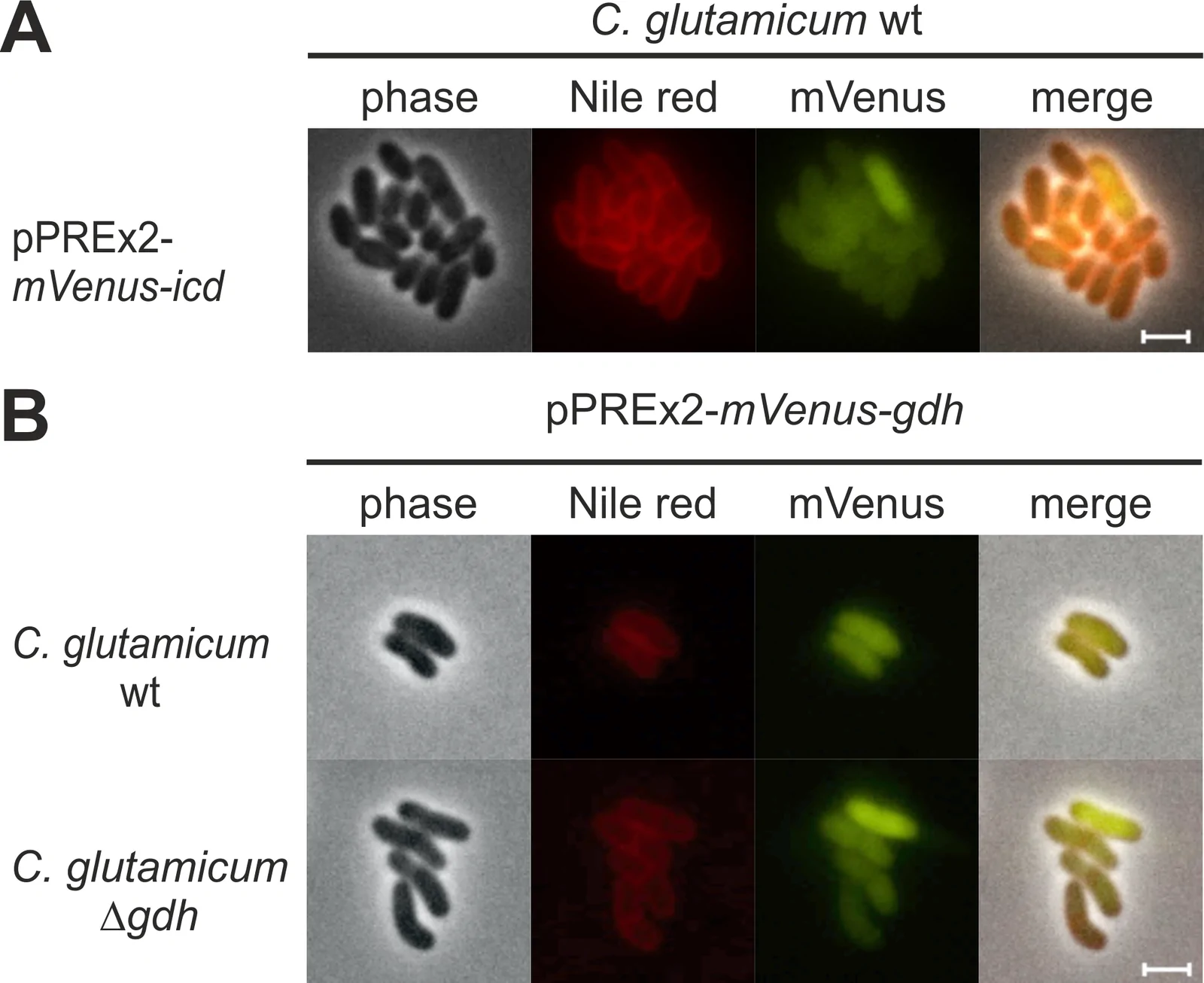
Localization of isocitrate dehydrogenase and glutamate dehydrogenase in *C. glutamicum*. (**A**) Exemplary images of *C. glutamicum* pPREx2-*mVenus-icd*. (**B**) Exemplary images of *C. glutamicum* pPREx2-*mVenus-gdh* and *C. glutamicum* ∆*gdh* pPREx2-*mVenus-gdh*. Cells were grown in CGXII medium with 2% (wt/vol) glucose. Cell membranes were stained using Nile red, and images were taken using an Axio imager M2 microscope. Scale bars represent 2 µm.

The cellular localization of the hybrid PDH-ODH complex triggered the question whether an even larger assembly of TCA cycle and ammonium assimilation enzymes is formed at the poles. The enzymes catalyzing the synthesis of 2-oxoglutarate and its reductive amination to L-glutamate, isocitrate dehydrogenase (Icd), and glutamate dehydrogenase (Gdh) were selected as candidates to test this possibility. We constructed the plasmids pPREx2-*mVenus-icd* and pPREx2-*mVenus-gdh* coding for N-terminal fusions of Icd or Gdh with mVenus and analyzed the localization of the fusion proteins in *C. glutamicum* wt and, in case of the Gdh construct, additionally in a Δ*gdh* strain. As shown in Fig. 5, both mVenus-Icd and mVenus-Gdh produced uniform fluorescence signals in the cytoplasm, suggesting that the localization at the poles and at mid-cell is a specific characteristic of the PDH-ODH complex.

## DISCUSSION

In this study, we analyzed the intracellular localization of the four subunits of the hybrid PDH-ODH complex, of the ODH inhibitor protein OdhI, and of isocitrate dehydrogenase and glutamate dehydrogenase of *C. glutamicum* using fusions with the fluorescent proteins mVenus or mCherry. In the initial experiments with an OdhI-mVenus fusion, we surprisingly observed bright fluorescent spots at the cell poles and, in longer cells, sometimes one or two further spots at mid-cell. These distinct spots were formed both after plasmid-based expression of *odhI-mVenus* and in a *C. glutamicum::odhI-mVenus* integration strain (Fig. 1), showing that the spots are not caused as an artefact of plasmid-based expression, e.g., by inclusion body formation due to overexpression. It rather appeared that the genomic expression of *odhI-mVenus* from its native promoter was even stronger than plasmid-based expression. Furthermore, we confirmed that the OdhI-mVenus protein is still functional in inhibiting ODH activity, as plasmid-based expression of *odhI-mVenus* in *C. glutamicum* ∆*pknG*∆*odhI* elicited a growth defect on glutamine agar plates similar to expression of native *odhI* (Fig. S3).

Besides the bright fluorescent spots, cells expressing *odhI-mVenus* also showed a weak fluorescence signal evenly distributed in the cell, suggesting that only a fraction of OdhI-mVenus is involved in the formation of the fluorescent spots, while another fraction is spread throughout the cytosol. Studies with mutant strains indicated that the OdhI fraction forming the spots represents unphosphorylated OdhI bound to the OdhA subunit of the ODH complex, whereas the fraction evenly distributed in the cytoplasm represents unbound phosphorylated OdhI: (i) The lack of one or several STPKs involved in OdhI phosphorylation did not prevent OdhI spot formation but even increased the number of spots per cell. (ii) The use of OdhI variants in which one or both phosphorylation sites, T14 and T15, were exchanged by alanine did not prevent spot formation. (iii) In the phosphatase-deficient mutant Δ*ppp*, in which almost all OdhI proteins are present in a phosphorylated state, spot formation was almost completely prevented. (iv) In a Δ*odhA* mutant, no spot formation was observed, and the OdhI-mVenus fluorescence was evenly distributed in the cytoplasm.

As suggested by the localization studies with OdhI, an OdhA-mCherry fusion formed fluorescent spots at the poles, too, and these spots perfectly co-localized with the OdhI-mVenus spots (Fig. 3). Also, the other three proteins of the PDH-ODH complex, AceE, AceF, and Lpd, formed fluorescent spots at the cell poles when fused with mVenus (Fig. 4A). Furthermore, AceE-mCherry, AceF-mCherry, and mCherry-Lpd fusion proteins showed co-localization with OdhA in *C. glutamicum::odhA-mVenus* (Fig. 4B). These results indicate that the entire PDH-ODH complex is located at the poles or at mid-cell. In contrast, fluorescent fusion proteins of isocitrate dehydrogenase as another TCA cycle enzyme and of glutamate dehydrogenase as an enzyme competing with ODH for the substrate 2-oxoglutarate showed a homogenous distribution in the cytoplasm (Fig. 5). The polar localization is, therefore, a specific feature of the PDH-ODH complex. The integration strains *C. glutamicum::odhI-mVenus*, *C. glutamicum::odhA-mVenus*, *C. glutamicum::aceE-mVenus*, and *C. glutamicum::aceF-mVenus* showed comparable growth as *C. glutamicum* wt, suggesting that an active PDH-ODH complex was formed by the fusion constructs (Fig. S4A). The specific fluorescence of these strains, which should reflect their native protein levels, suggests that OdhI is formed in twofold excess of OdhA and that AceE and AceF are present at slightly higher levels than OdhA (Fig. S4B).

A possible reason for the specific localization of the PDH-ODH complex could be an interaction of one of the subunits with another protein that is located at the poles or at the division site. In this case, the absence of this subunit should abolish the observed localization. We excluded AceE and OdhA as candidates, as in the Δ*aceE* and Δ*odhA* mutants, the other tested proteins of the complex were still located at the poles. Unfortunately, we were not able to obtain Δ*aceF* and Δ*lpd* mutants and, therefore, could not test their relevance for the polar localization of the residual subunits. The primary structures of the PDH-ODH subunits do not contain extra domains hinting at an interaction with a yet unknown protein, but this does not exclude such an interaction. Another mechanism which was found to cause specific intracellular localization of proteins is liquid-liquid phase separation (LLPS). This formation of biomolecular condensates is often related to protein DNA/RNA interactions, such as the clustering of bacterial RNA polymerases (37) or the specific localization observed for DEAD-box helicases in *E. coli* (38) and ribonuclease E in *Caulobacter crescentus* (39). Treatment with 1,6-hexanediol has emerged as an LLPS assay and was previously used to distinguish between liquid-like condensates of LLPS and solid-like condensates of bacteria, yeast, and mammalian cells, even if the mechanism is not completely understood yet and potential side effects need to be excluded (37, 40 – 42). We analyzed the influence of 1,6-hexanediol on the formation of the fluorescent spots caused by AceF-mVenus and did not observe an effect under the tested conditions (Fig. S5), suggesting that LLPS is not the most likely explanation for the distinct localization of the PDH-ODH hybrid complex.

An alternative explanation for the distinct polar spots is nucleoid exclusion (43, 44) caused by the unique structural features of the hybrid PDH-ODH complex that might cause the formation of few large assemblies comprising the vast majority of all four subunits. Like many other bacteria, *C. glutamicum* contains nucleoids (45, 46) that probably affect the dynamics and localization of large cellular components, such as ribosomes, whose diffusion is impeded by the DNA meshwork of the nucleoid (47, 48). This potential explanation is supported by the observation that the fluorescent spots formed by the PDH-ODH subunits were mainly visible in the DNA-free regions, as observed by SYTOX Orange DNA staining in the integration strains *C. glutamicum::odhI-mVenus*, *C. glutamicum::odhA-mVenus*, *C. glutamicum::aceE-mVenus*, and *C. glutamicum::aceF-mVenus* (Fig. 4C). Nucleoid exclusion as cause of the polar localization of the PDH-ODH complex might also explain the observed correlation between the number of OdhI-mVenus spots and cell length (Fig. 1 and 2). Prolonged cells might possess more nucleoid-free space that is available for the assembly of large complexes, such as the PDH-ODH complex, enabling the formation of a higher number of these assemblies per cell.

The native structure of the hybrid PDH-ODH complex is not known yet, but it certainly differs from the structures reported for non-actinobacterial PDH and ODH complexes, such as PDH of *E. coli*, where the core is formed by eight E2p trimers to which E1p dimers and E3 dimers are bound via the peripheral subunit binding domain (PSBD) of E2p. In actinobacteria, E2p (AceF) also forms a trimer, but a phenylalanine-containing three-residue-insertion in the C-terminal helix prevents higher order oligomerization (17). Biochemical studies with reconstituted complexes suggested that two AceF trimers form the core of the hybrid complex and are stably associated with Lpd dimers binding to the PSBD domains of AceF (16, 49). OdhA was proposed to form hexamers and a smaller oligomer that readily associated with the AceF-Lpd subcomplex, whereas AceE appeared to have a lower affinity for this subcomplex. For the reconstituted ODH complex, a size of 940 kDa was estimated (16). Recent structural studies confirmed that OdhA is an 800-kDa homohexamer that folds into a three-blade propeller shape (20). Although the minimal hybrid PDH-ODH complex with about 1 MDa appears to be much smaller than the separate PDH and ODH complexes of known structure (e.g., approximately 4.5 MDa for *E. coli* PDH), its specific structural organization might enable the formation of large three-dimensional networks which are excluded from the nucleoid, explaining the formation of mostly only two to four large assemblies located at the poles or at mid-cell of larger cells. As the lack of OdhA or AceE did not prevent the localization of the residual subunits at the poles, the stable AceF-Lpd subcomplex could be sufficient for the assembly of such networks. As Lpd forms dimers, a speculation could be that each of the monomers binds to PSBD domains of different AceF complexes and thereby form a three-dimensional network. Further structural analysis is required to prove or disprove the formation of PDH-ODH networks.

Bioinformatic analysis revealed that OdhA homologs are present in many genera of actinobacteria and always linked to the presence of an E2p protein with the three-residue insertion preventing assembly of the trimers (17). Therefore, hybrid PDH-ODH complexes are probably formed in all these species, and a similar localization as in *C. glutamicum* caused by nucleoid exclusion appears likely. Of course, a structural view on this complex would be the obvious next target but might be hampered by the high flexibility of interdomain linkers within AceF. The observation of a defined spatial localization of an enzyme complex catalyzing two key reactions of central metabolism poses questions regarding possible consequences for the availability of substrates and products within the cell and if further enzyme complexes of bacteria show a similar behavior.

## MATERIALS AND METHODS

### Bacterial strains, media, and culture conditions

All bacterial strains and plasmids used in this work are listed in Table 1. *Escherichia coli* cells were cultivated at 37°C in lysogeny broth (LB) (50) or on LB agar plates (Carl Roth, Karlsruhe, Germany). *C. glutamicum* strains were cultivated at 30°C in brain-heart infusion medium (BHI; Difco Laboratories, Detroit, USA) or in CGXII medium with 2% (wt/vol) glucose (51) containing 30 mg L^−1^ 3,4-dihydroxybenzoate as iron chelator. A 15 g L^−1^ agar was added to prepare the respective solid media. Kanamycin was added at concentrations of 25 mg L^−1^ (*C. glutamicum*) or 50 mg L^−1^ (*E. coli*) to maintain plasmid stability. In case of the *C. glutamicum* ∆*aceE* strain, the culture medium was supplemented with 2 g L^−1^ acetate.

**TABLE 1:** Bacterial strains and plasmids used in this study

Strain or plasmid	Description	Reference or source
*C. glutamicum* strains		
ATCC13032	Biotin-auxotrophic wild-type strain	DSMZ
Δ*odhI*	Wild-type derivative with in-frame deletion of *odhI* (cg1630)	(14)
Δ*pknG*Δ*odhI*	Wild-type derivative with in-frame deletion of *pknG* (cg3046) and *odhI* (cg1630)	(14)
Δ*pknA*Δ*pknL*Δ*pknG*	Wild-type derivative with in-frame deletion of *pknA* (cg0059), *pknL* (cg2388), and *pknG* (cg3046)	(25)
Δ*pknB*Δ*pknL*Δ*pknG*	Wild-type derivative with in-frame deletion of *pknB* (cg0057), *pknL* (cg2388), and *pknG* (cg3046)	(25)
Δ*ppp*	Wild-type derivative with in-frame deletion of *ppp* (cg0062)	(23)
Δ*odhA*	Wild-type derivative with in-frame deletion of *odhA* (cg1280)	This work
Δ*aceE*	Wild-type derivative with in-frame deletion of *aceE* (cg2466)	(52)
Δ*gdh*	Wild-type derivative with in-frame deletion of *gdh* (cg2280)	(53)
*::odhI-mVenus*	Wild-type derivative with in-frame insertion of a linker sequence (GGTACCGCAGCG) and *mVenus* encoding for a C-terminal fusion of OdhI(Cg1630)-mVenus	This work
*::odhA-mVenus*	Wild-type derivative with in-frame insertion of a linker sequence (GGTACCGCAGCG) and *mVenus* encoding for a C-terminal fusion of OdhA(Cg1280)-mVenus	This work
*::aceE-mVenus*	Wild-type derivative with in-frame insertion of a linker sequence (GGTACCGCAGCG) and *mVenus* encoding for a C-terminal fusion of AceE(Cg2466)-mVenus	This work
*::aceF-mVenus*	Wild-type derivative with in-frame insertion of a linker sequence (GGTACCGCAGCG) and *mVenus* encoding for a C-terminal fusion of AceF(Cg2421)-mVenus	This work
*M. tuberculosis* strains		
H37Rv	Chromosomal DNA was used as template for amplification of *garA* (Rv1827)	DSMZ
*E. coli* strains		
DH5α	F- supE44 *ΔlacU*169 (Φ80*lacZΔM15*) *hsdR17 recA1 endA1 gyrA96 thi-1 relA1*	(54)
Plasmids		
pK19*mobsacB*	Kan^R^; suicide vector for allelic exchange in *C. glutamicum; oriV*_*E.c.*_ *oriT sacB*	(55)
pK19*mobsacB*-∆*odhA*	Kan^R^; pK19*mobsacB* derivative containing PCR products covering the up- and downstream regions of the *odhA* (cg1280) gene	This work
pK19*mobsacB*-*odhI-mVenus*	Kan^R;^ pK19*mobsacB* derivative containing PCR products covering the up- and downstream regions of the *odhI* (cg1630) stop codon and the mVenus encoding sequence	This work
pK19*mobsacB-odhA-mVenus*	Kan^R^; pK19*mobsacB* derivative containing PCR products covering the up- and downstream regions of the *odhA* (cg1280) stop codon and the mVenus encoding sequence	This work
pK19*mobsacB*-*aceE-mVenus*	Kan^R^; pK19*mobsacB* derivative containing PCR products covering the up- and downstream regions of the *aceE* (cg2466) stop codon and the mVenus encoding sequence	This work
pK19*mobsacB*-*aceF-mVenus*	Kan^R^; pK19*mobsacB* derivative containing PCR products covering the up- and downstream regions of the *aceF* (cg2421) stop codon and the mVenus encoding sequence	This work
pDHL-*mVenus*	Kan^R^ , pDHL1029 derivative, used for amplification of mVenus encoding sequence	(56)
pPREx2	Kan^R^; pPBEx2 derivative (P*_tacI_, lacI^q^, ori_C.g_* from pBL1.; *ori_E.c_*_._ ColE1 from pUC18), with a consensus RBS (AAGGAG) for *C. glutamicum*	(57)
pPREx2-*mVenus*	Kan^R^; pPREx2 derivative carrying the mVenus encoding sequence	This work
pPREx2-*odhI-mVenus*	Kan^R^; pPREx2 derivative carrying the OdhI (Cg1630) and mVenus encoding sequences fused by a linker sequence (GGTACCGCAGCG)	This work
pPREx2*-garA-mVenus*	Kan^R^; pPREx2 derivative carrying the GarA (Rv1827) and mVenus encoding sequences fused by a linker sequence (GGTACCGCAGCG)	This work
pPREx2-*target-mVenus*	Kan^R^; pPREx2 derivative carrying a linker sequence (GGTACCGCAGCG) and the mVenus encoding sequence to enable C-terminal mVenus fusions to different targets	This work
pPREx2-*mVenus-target*	Kan^R^; pPREx2 derivative carrying the mVenus encoding sequence and a linker sequence (GGTACCGCAGCG) to enable N-terminal mVenus fusions to different targets	This work
pPREx2-*aceE-mVenus*	Kan^R^; pPREx2*-target-mVenus* derivative carrying the AceE (Cg2466) encoding sequence	This work
pPREx2-*aceF-mVenus*	Kan^R^; pPREx2-*target-mVenus* derivative carrying the AceF (Cg2421) encoding sequence	This work
pPREx2-*mVenus-lpd*	Kan^R^; pPREx2-*mVenus-target* derivative carrying the Lpd (Cg0441) encoding sequence	This work
pPREx2-*mVenus-icd*	Kan^R^; pPREx2-*mVenus-target* derivative carrying the Icd (Cg0766) encoding sequence	This work
pPREx2-*mVenus-gdh*	Kan^R^; pPREx2-*mVenus-target* derivative carrying the Gdh (Cg2280) encoding sequence	This work
pPREx2*-odhI-mCherry*	Kan^R^; pPREx2 derivative carrying the OdhI (Cg1630) and mCherry encoding sequence fused by a linker sequence (GGTACCGCAGCG)	This work
pPREx2-*odhA-mCherry*	Kan^R^; pPREx2 derivative carrying the OdhA (Cg1280) and mCherry encoding sequence fused by a linker sequence (GGTACCGCAGCG)	This work
pPREx2-*aceE-mCherry*	Kan^R^; pPREx2-*odhI-mCherry* derivative carrying the AceE (Cg2466) encoding sequence, *odhI* was removed by restriction digestion using NdeI and KpnI	This work
pPREx2-*aceF-mCherry*	Kan^R^; pPREx2-*odhI-mCherry* derivative carrying the AceF (Cg2421) encoding sequence, *odhI* was removed by restriction digestion using NdeI and KpnI	This work
pPREx2-*mCherry-target*	Kan^R^; pPREx2 derivative carrying the mCherry encoding sequence and a linker sequence (GGTACCGCAGCG) to enable N-terminal mCherry fusions to different targets	This work
pPREx2-*mCherry-lpd*	Kan^R^; pPREx2-*mCherry-target* derivative carrying the Lpd (Cg0441) encoding sequence	This work

### Standard recombinant DNA work and construction of deletion mutants

Standard methods such as PCR and plasmid restriction were carried out according to established protocols (58), and all oligonucleotides used are listed in Table S1. Plasmids were constructed by Gibson assembly (59). Oligonucleotides were ordered from and DNA sequencing was performed by Eurofins Genomics (Ebersberg, Germany). Transformation of *E. coli* was performed following a standard protocol (54), and *C. glutamicum* transformation was performed by electroporation (60). *C. glutamicum* deletion or integration mutants were constructed by double homologous recombination using pK19*mobsacB*-based plasmids as described previously (61). Oligonucleotides annealing upstream and downstream of the target genes were used to confirm genomic deletions or integrations by colony PCR.

### Fluorescence microscopy

Prior to analysis by fluorescence microscopy, the different *C. glutamicum* strains were grown overnight at 30°C and 170 rpm in BHI medium, diluted 1:50 to inoculate a main culture in CGXII medium with 2% (wt/vol) glucose, and incubated for 3–4 h under the same conditions. For membrane staining, cells were harvested by centrifugation (5 min, 4000 × *g*) and resuspended in phosphate-buffered saline (PBS; 137 mM NaCl, 2.7 mM KCl, 10 mM Na_2_HPO_4_, 1.8 mM KH_2_PO_4_, pH 7.4) containing 250 ng ml^−1^ Nile red followed by 10-min incubation in the dark. For DNA staining, 500 nM SYTOX Orange (Thermo Fisher Scientific, Waltham, MA, USA) was added, and the cultures were further incubated for 10 min in the same cultivation conditions. Afterwards, cells were collected by centrifugation at 5,000 × *g*, washed once, and resuspended in PBS. Afterwards, cells were immobilized on glass slides with agar pads [0.9% (wt/vol) NaCl, 1.5% (wt/vol) agarose] and analyzed using an Axio imager M2 microscope equipped with AxioCam ICc 3 and a Zeiss Plan-Apochromat 100×/1.4 oil Ph3 objective and an HXP 120 C lightning unit (Carl Zeiss). For detection of mVenus fluorescence, the filter 46 HE (λex 500/25 nm, λem 535/30 nm) and, in case of mCherry, Nile red, or SYTOX Orange, the filter 43 HE (λex 545/25 nm, λem 605/70 nm) were used. Image processing took place using the AxioVision SE64 Rel. software v. 4.8.2 (Carl Zeiss).

### Statistics

For statistical analysis of the fluorescent spots and the cell size, at least 100 cells were analyzed of each strain. Cell size was measured using the AxioVision SE64 Rel. software v. 4.8.2 (Carl Zeiss), and fluorescent spots were counted manually. Dividing cells were counted as individual cells if the Nile red staining showed a new membrane at the division site. Data analysis was carried out using GraphPad Prism 9 (GraphPad, San Diego, USA). The frequency of different cell length was calculated using Graphpad Prism 9 frequency analysis with a bin width of 0.5.

## SUPPLEMENTAL MATERIAL

Supplemental file 1 spectrum.02668-23-s0001.pdf Table S1 and Fig. S1 to S5

ASM does not own the copyrights to Supplemental Material that may be linked to, or accessed through, an article. The authors have granted ASM a non-exclusive, world-wide license to publish the Supplemental Material files. Please contact the corresponding author directly for reuse.
