# Supplementary material for: Cellular localization of the hybrid pyruvate/2-oxoglutarate dehydrogenase complex in the actinobacterium Corynebacterium glutamicum
Source: Microbiol Spectr. 2023 Sep 27;11(5):e02668-23. doi: 10.1128/spectrum.02668-23 (PMC10581250; doi:10.1128/spectrum.02668-23)
Supplement: Supplemental file 1 — Table S1 and Fig. S1 to S5 [file spectrum.02668-23-s0001.pdf]

Supplementary information

Cellular localization of the hybrid pyruvate/2-oxoglutarate dehydrogenase complex in the actinobacterium *Corynebacterium glutamicum*

Lea Sundermeyer[1], Jan-Gerrit Folkerts[1], Benita Lückel[1], Christina Mack[1], Meike Baumgart[1], and Michael Bott[1,2*]

[1]IBG-1: Biotechnology, Institute of Bio- and Geosciences, Forschungszentrum Jülich, 52425 Jülich, Germany

[2]Bioeconomy Science Center (BioSC), Forschungszentrum Jülich, 52425 Jülich, Germany

*Corresponding author: Prof. Dr. Michael Bott, IBG-1: Biotechnology, Institute of Bio- and Geosciences, Forschungszentrum Jülich, 52425 Jülich, Germany

Phone +49 2461 613294; Email m.bott@fz-juelich.de

Table S1. Oligonucleotides used in this study

Name	Sequence
Construction of pK19mobsacB-Δ*odhA*	
ΔodhA_1	CAGGTCGACTCTAGAGGAAACATTGGCTACGGATGC
ΔodhA_2	GGCAGGTACTCGCCTCTTTTC
ΔodhA_3	AAGAGGCGAGTACCTGCCTAAGTCTTTATAGTCCTGCACTAGC
ΔodhA_4	AAAACGACGGCCAGTGAATTATCGGCTGGGTGGATTTC
Construction of pK19mobsacB-*odhI-mVenus*	
odhI-mVenus_I1	CGACGTTGTAAAACGACGGCCAGTGGACAACCCACTTGCGGG
odhI-mVenus_I2	CTCCTTTGCTAGCCGCTGCGGTACCCTCAGCAGGGCCTGCG
odhI-mVenus_I3	GGTACCGCAGCGGCTAGCAAAGGAGAAGAACTTTTCACTG
odhI-mVenus_I4	TAGGAAGTGTTTTTATTTGTAGAGCTCATCCATGCCATG
odhI-mVenus_I5	GCTCTACAAATAAAAACACTTCCTAGGAAAGTTCTTTGCAG
odhI-mVenus_I6	GCATGCCTGCAGGTCGACTCTAGAGCCAAAGGCTCCGAGGAGG
Construction of pK19mobsacB-*odhA-mVenus*	
odhA-mVenus_I1	TTATTTGTAGAGCTCATCCATGC
odhA-mVenus_I2	GACGAGGCTTTCGAGGCTGGTACCGCAGCGGCTAGCAAAGGAGAAGAAC
odhA-mVenus_I3	CAGGTCGACTCTAGAGGAATCGGCTGGGTGGATTTC
odhA-mVenus_I4	GATGAGCTCTACAAATAAGTCTTTATAGTCCTGCACTAGC
odhA-mVenus_I5	AGCCTCGAAAGCCTCGTC
odhA-mVenus_I6	AAAACGACGGCCAGTGAATTCGTCCACTGGTTATCTTC
Construction of pK19mobsacB-*aceE-mVenus* (template: pPREx2-aceE-mVenus and *C. glutamicum* gDNA)	
aceE-mVenus_I1	GTAAAACGACGGCCAGTGGAAGGCACCGGCCATGAGG
aceE-mVenus_I2	TTATTTGTAGAGCTCATCCATGC
aceE-mVenus_I3	ATGGATGAGCTCTACAAATAAATCACCTCAAGGGACAGAT
aceE-mVenus_I4	TGCAGGTCGACTCTAGAGCTTTGCACTGACCTCGAAGCG
Construction of pK19mobsacB-*aceF mVenus* (template: pPREx2-aceF-mVenus and *C. glutamicum* gDNA)	
aceF-mVenus_I1	GTAAAACGACGGCCAGTGGCAGTTGTCGAGGCTTTG
aceF-mVenus_I2	TTATTTGTAGAGCTCATCCATGC
aceF-mVenus_I3	ATGGATGAGCTCTACAAATAAGATCTCTGCAAGTTAAAACC
aceF-mVenus_I4	TGCAGGTCGACTCTAGAGCCAGCGCCGGCAACACCCATAT
Construction of pPREx2-*mVenus*	
mVenus fw	AGAAGGAGATATACATATGGCTAGCAAAGGAGAAGAAC
mVenus rv	AAACGACGGCCAGTGAATTCTTATTTGTAGAGCTCATCCATGC

Construction of pPREx2-*odhI-mVenus*

pPREx2_odhI fw	AGAAGGAGATATACATATGAGCGACAACAACGGCACCC
odhI rv	CTCAGCAGGGCCTGCGAG
odhI-mVenus fw	GCAGGCCCTGCTGAGGGTACCGCAGCGGCTAGCAAAGGAGAAGAAC
mVenus_pPREx2 rv	AAACGACGGCCAGTGAATTCTTATTTGTAGAGCTCATCCATGC

Construction of pPREx2-*garA-mVenus*

pPREx2_garA fw	TGCAGAAGGAGATATACAtATGACGGACATGAACCCGGATATTG
garA rv	GCTAGCCGCTGCGGTACCCGGGCCCCCGGTACTCCCGTC
garA-mVenus fw	GGTACCGCAGCGGCTAGCAAAGGAGAAGAAC
mVenus_pPREx2 rv	AAACGACGGCCAGTGAATTCTTATTTGTAGAGCTCATCCATGC

Construction of pPREx2-*target-mVenus*

linker-mVenus fw	ATATACATATGACCTGAGGGTACCGCAGCGGTGAGCAAGGGCGAGGAGCT TTTCACTGGAGTTGTC
mVenus rv	AAACGACGGCCAGTGAATTCTTATTTGTAGAGCTCATCCATGC

Construction of pPREx2 *-mVenus-target*

mVenus fw	AGAAGGAGATATACATATGGCTAGCAAAGGAGAAGAAC
mVenus-linker rv	AAACGACGGCCAGTGAATTCCGCTGCGGTACCTTTGTAGAGCTCATCCATG C

Construction of pPREx2-*aceF-mVenus* and pPREx2-*aceF-mCherry*

aceF fw	TGCAGAAGGAGATATACAtATGGCGTTCTCCGTAGAGATG
aceF rv	TGCTCACCGCTGCGGTACCGAGCTGCAGATCGCCTTCG

Construction of pPREx2-*aceE-mVenus* and pPREx2-*aceE-mCherry*

aceE fw	TGCAGAAGGAGATATACAtATGGCCGATCAAGCAAAACTTGGTGG
aceE rv	TGCTCACCGCTGCGGTACCTTCCTCAGGAGCGTTTGG

Construction of pPREx2-*mVenus-lpd*

lpd fw	TGAGCTCTACAAAGGTACCGCAGCGGTGACTGAACATTATGACGTAG
lpd rv	AAACGACGGCCAGTGAATTCCTAGAAGTTGATCATGTGTCCAGAG

Construction of pPREx2-*mVenus-icd*

icd fw	TGAGCTCTACAAAGGTACCGCAGCGATGGCTAAGATCATCTGGAC
icd rv	AAACGACGGCCAGTGaattcTTACTTCTTCAGTGCGTCAAC

Construction of pPREx2-*mVenus-gdh*

gdh fw	TGAGCTCTACAAAGGTACCGCAGCGACAGTTGATGAGCAGGTC

gdh rv	AAACGACGGCCAGTGaattcTTAGATGACGCCCTGTGC

Construction of pPREx2-*odhI-mCherry*

pPREx2_odhI fw	AGAAGGAGATATACATATGAGCGACAACAACGGCACCC
odhI-mcherry rv	TCCTCCTCGCCCTTGCTCACCGCTGCGGTACCCTCAGCAGGGCCTGCGAG
mCherry fw	GTGAGCAAGGGCGAGGAG
mCherry_pPREx2 rv	AAACGACGGCCAGTGAATTCTTACTTGTACAGCTCGTC

Construction of pPREx2-*odhA-mCherry*

pPREx2_odhA fw	GCATGCCTGCAGAAGGAGATATACATGTGAGCAGCGCTAGTACTTTCGG
odhA rv	AGCCTCGAAAGCCTCGTC
odhA-mCherry fw	GCTTATCGACGAGGCTTTCGAGGCTGGTACCGCAGCGG
mCherry_pPREx2 rv	AAACGACGGCCAGTGAATTCTTACTTGTACAGCTCGTC

Construction of pPREx2-*mCherry-target* (PCR template pPREx2-mCherry)

mCherry-target fw	TTGCATGCCTGCAGAAGGAG
mCherry-target rv	AAACGACGGCCAGTGAATTCCGCTGCGGTACCCTTGTACAGCTCGTCCATGCC

Construction of pPREx2-*mCherry-lpd*

mCherry-lpd fw	CGAGCTGTACAAGGGTACCGCAGCGGTGACTGAACATTATGACGTAG
lpd rv	AAACGACGGCCAGTGAATTCCTAGAAGTTGATCATGTGTCCAGAG

Confirmation of gene deletion and integration by colony-PCR

DodhA_fw	AGGACGCCCAAACAACAG
DodhA_rv	AATGAAGGTGCCGTGAAAG
DaceE_fw	ACAACAATTGCCAGGAAGC
DaceE_rv	TGTCTACCAGGCAGATCAG
IodhI_fw	CACGAGGGTGCCCATTTG
IodhI_rv	GTCAGTGAGGCGAGTAATC
IodhA_fw	CACTCTTCCGCACGTATC
IodhA_rv	AGGACGCCCAAACAACAG
IaceE_fw	AGCCAGCTGAGCCAGAAG
IaceE_rv	AGATTTCCATCGACGGCAAC
IaceF_fw	ACCACTCAGAAGGTCAAC
IaceF_rv	TGCAACACCACGAGTTATTC

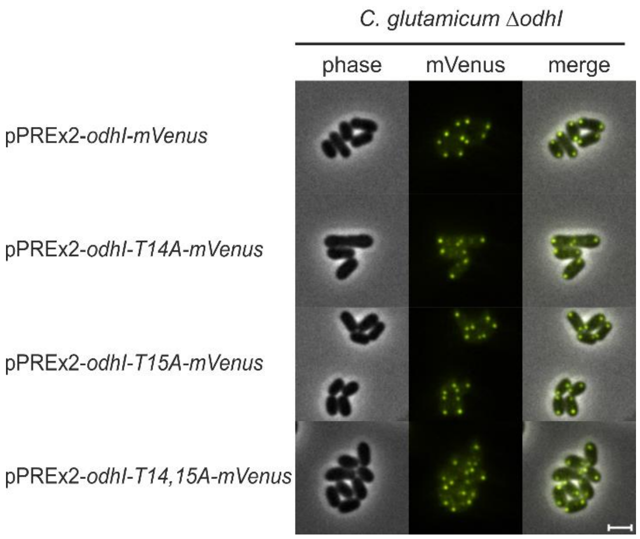

Fig. S1. Influence of the mutation of OdhI phosphorylation sites on OdhI-mVenus localization. Exemplary images of *C. glutamicum* Δ*odhI* carrying the expression plasmids pPREx2-*odhI*-*mVenus*, pPREx2-*odhI*-T14A-*mVenus*, pPREx2-*odhI*-T15A-*mVenus*, and pPREx2-*odhI*-T14,15A-*mVenus*. All strains were grown in CGXII medium with 2 % (w/v) glucose and images were taken using an Axio imager M2 microscope. Scale bar represents 2 µm.

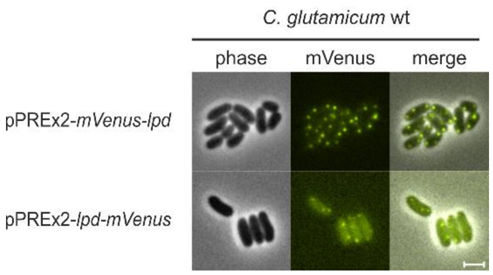

Fig. S2. Comparison of the effect of the fusion of mVenus to the C- and N-terminus of Lpd on the formation of fluorescencent spots.

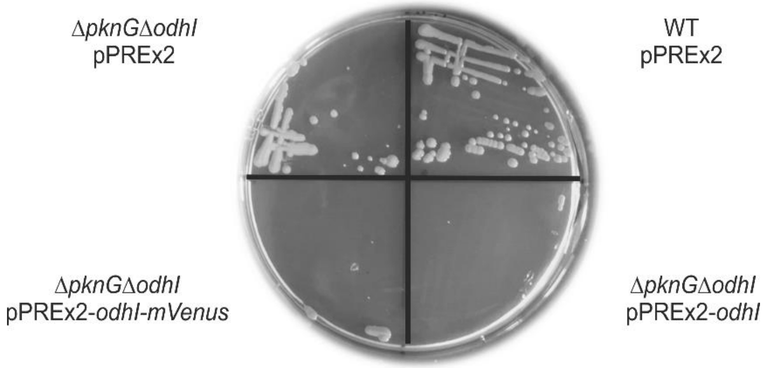

Fig. S3. Growth of the indicated *C. glutamicum* strains on agar plates containing L-glutamine as sole carbon and nitrogen source to test for OdhI-mVenus functionality. Like native OdhI, the presence of the OdhI-mVenus fusion protein in *C. glutamicum ΔpknGΔodhI* inhibited growth on glutamine, indicating that the OdhI-mVenus protein is functional in inhibiting ODH activity.

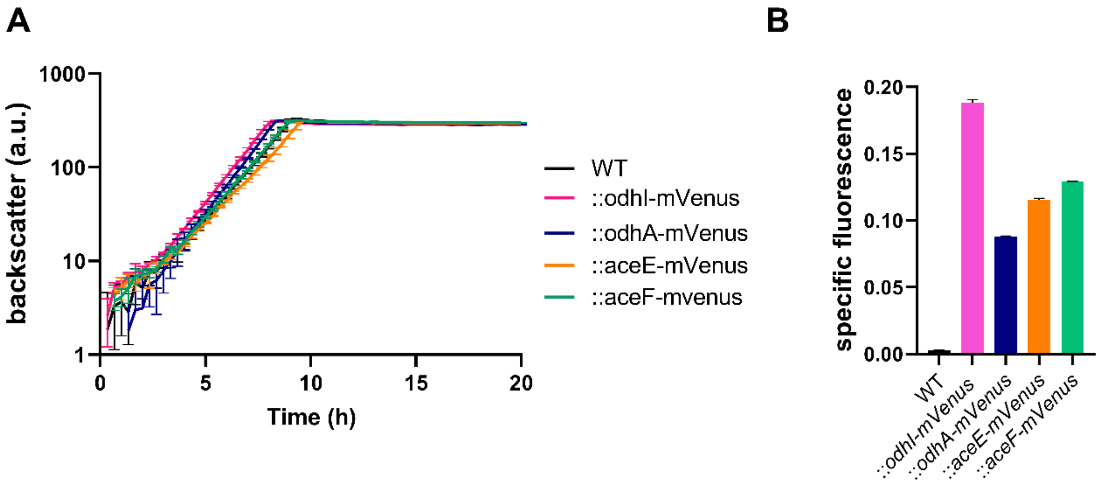

Fig. S4. Growth (A) and specific fluorescence after 20 h cultivation (B) of *C. glutamicum* wt and the indicated mVenus integration strains. The strains were cultivated in biological triplicates in CGXII medium with 2% (w/v) glucose at 30°C, 1200 rpm in a Biolector microcultivation system. Mean values and standard deviations are shown.

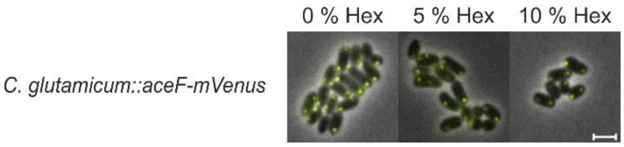

Fig. S5. Exemplary images of *C. glutamicum::aceF-mVenus* incubated either without 1,6-hexanediol (Hex) or with 5% (w/v) or 10% (w/v) 1,6-hexanediol for 5 min under standard cultivation conditions. Cells were grown in CGXII medium with 2% (w/v) glucose and images were taken using an Axio imager M2 microscope. Scale bar represents 2 µm.
